# Percutaneous Renal Cyst Ablation and Review of the Current Literature

**DOI:** 10.1089/cren.2015.0013

**Published:** 2016-02-01

**Authors:** Devang Desai, Sunny Modi, Matthew Pavicic, Melissa Thompson, John Pisko

**Affiliations:** ^1^ Urology Department, Gold Coast Hospital, Gold Coast, Australia.; ^2^ Urology Department, Greenslopes Private Hospital, Brisbane, Australia.

## Abstract

Renal cysts are common and most often are discovered incidentally, but may require intervention if associated with pain, hypertension, or hematuria. Minimally invasive treatment options are preferred with numerous modalities available, including renal cyst ablation. This case report of a 61-year-old female describes the effective percutaneous drainage and endoscopic ablation of a simple parapelvic renal cyst for management of symptomatic renal calculus. Current literature regarding this surgical intervention and alternative methods is discussed.

## Introduction

Renal cysts are a commonly encountered finding among the general population, often discovered incidentally as a result of radiological investigation.^1,2^ The incidence of simple cysts increases with age, and although mostly asymptomatic, they require intervention when associated with pain, hypertension, or hematuria.^2–4^ Treatment options for simple cysts continue to evolve with minimally invasive modalities now preferred.

## Materials and Methods

**FIG. 1.**
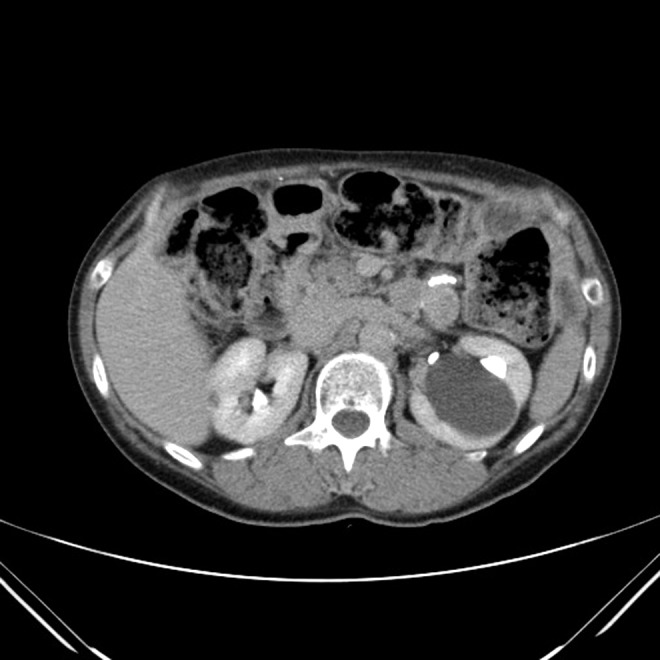
Large left central region renal cyst with adjacent 8 mm calculus in upper pole calix.

**FIG. 2.**
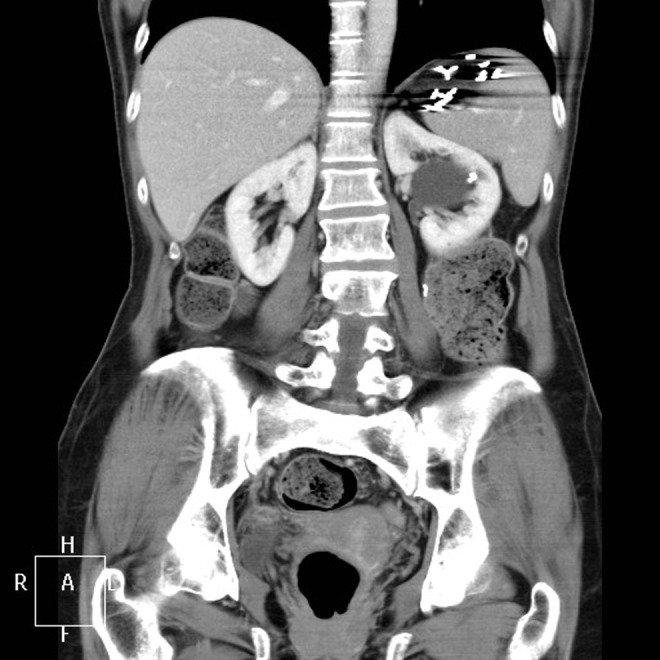
CT scan—coronal plane showing large renal cyst with renal calculus.

A 61-year-old female was referred to the urology service with recurrent left-sided flank pain. The patient's surgical history included intestinal volvulus requiring bowel resection, bariatric abdominal surgery, and nephrolithiasis. Her CT imaging revealed a single 8 mm stone in the upper pole calix of the left kidney as well as a large 5.5 cm Bosniak-I cyst of the left central region of the kidney (Figs. 1 and 2). The pain was thought to be a combination of the cyst and the stone. Distortion of the collecting system due to the cyst made it unamenable for ureteroscopy or extracorporeal shockwave lithotripsy. The cyst could be easily accessed percutaneously, with almost no parenchyma overlying. There was no preoperative renal scan performed.

**FIG. 3.**
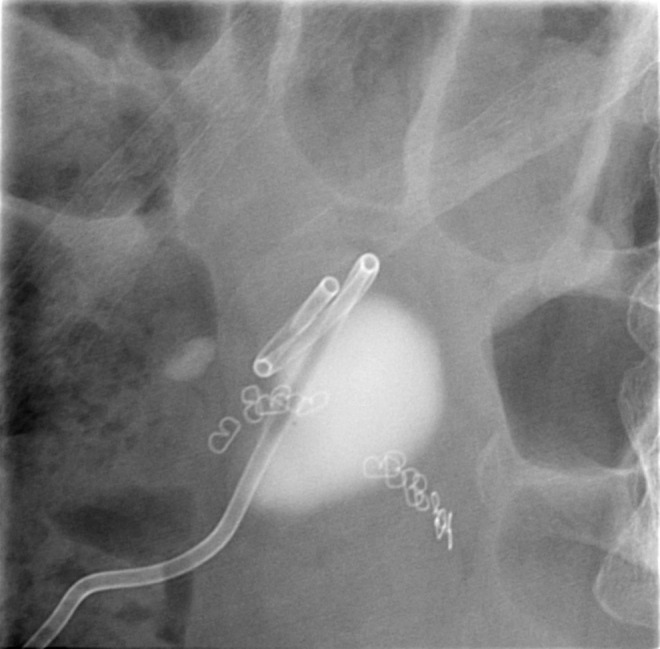
Pigtail catheter radiologically inserted into renal cyst cavity. Note staple line from previous bowel surgery.

**FIG. 4.**
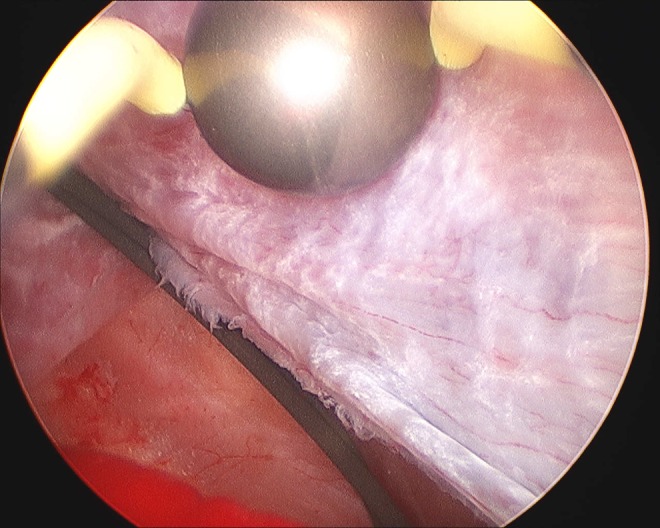
Renal cyst cavity ablation using 26F resectoscope and roller ball diathermy.

The patient underwent initial 8F pigtail catheter insertion in the cyst cavity the morning of the surgery under CT guidance in the radiology department (Fig. 3), and then, the tract was dilated under fluoroscopic guidance using a 30F Amplatz balloon over a guidewire under general anesthesia that afternoon. The cyst wall was ablated using a 26F resectoscope and roller ball diathermy (zero cut and 30 coagulation spray setting) without penetrating the cyst wall (Fig. 4). A 20F pigtail catheter was inserted into the cavity that was removed a week later without incident. The patient was discharged day 2. One month following cyst ablation, she underwent flexible ureteroscopy. Retrograde pyelogram showed no further distortion of the collecting system and easy access to the stone that was removed using a basket. Pain was partially relieved by treating the cyst and completely resolved after the stone was treated.

On follow-up at 3 and 5 months, there was no recurrence of the cyst on ultrasound imaging and the patient's symptoms were resolved.

## Discussion

Autopsy studies have suggested that more than 50% of persons older than 50 years of age have at least one simple renal cyst.^2,4^ The natural history of a simple renal cyst is for progression in size and number. Symptomatic cysts, which are variously reported as accounting for between 2% to 4% and 6% to 8% of simple cysts, may be treated by a variety of modalities.^4,5^ Modern approaches to renal cyst management include percutaneous aspiration, with or without sclerotherapy, and excision or marsupialization using laparoscopic, single port laparoscopic, ureteroscopic, and percutaneous approaches. Open surgery is rarely performed.^4–7^

Percutaneous cyst drainage alone is frequently undertaken but is associated with recurrence and reaccumulation of fluid in up to 80% of cases.^4^ Sclerotherapy using a variety of sclerosing agents, most commonly ethanol, has been used to reduce the rate of recurrence.^4,5^ Recurrence after aspiration and sclerosis, however, frequently occurs and is thought to be due to incomplete ablation of the cyst wall by the sclerosing agent. Surgical intervention of simple renal cysts has traditionally occurred using a transperitoneal or retroperitoneal laparoscopic approach depending on the cyst location.^8^ Laparoscopy has a success rate of 95% to 100% for managing simple renal cysts.^5^ Disadvantages of this technique include the requirement for multiple port sites, extensive dissection, and greater surgical experience relative to a percutaneous approach.^5,7^ A retrograde ureteroscopic approach has also been described.^5,7^ This offers a less invasive approach than laparoscopic and percutaneous management but lacks a robust body of evidence in support of its use. Its use is also limited to small cysts that are located near the collecting system.^7^. Surgical management of simple renal cysts using percutaneous access has previously been described in the literature.^5^ Techniques used using percutaneous access include ablation with cyst resection and percutaneous intrarenal marsupialization (endocystolysis).^3,5,7^

In our case, the entire cyst wall was visualized, and the cyst wall was ablated using low-voltage diathermy without penetrating the cyst wall. This is the first time the authors have treated a cyst of this size (5.5 cm Bosniak-I cyst). The cyst was initially treated, which partly relieved the patient's symptoms. Having got the access to the stone, ureteroscopic management of the stone was performed, which completely resolved the patient's symptoms. The authors felt that the patient's pain was from the cyst and that ablating the cyst would resolve the pain. It only resolved the pain partially and it also made the subsequent ureteroscopy easier to perform.

## Conclusion

Renal cysts can distort the collecting system with segmental obstruction. Stones developing in these segments are difficult to manage. Although this technique is not new, it is not reported being used in such a large renal cyst with complete resolution on follow-up. And given that it also provided easy access to treat the upper pole calix stone, which helped relieve the patient's symptoms completely, this combined technique offers a solution to this difficult problem.

## Abbreviation Used

CT: computed tomography
